# Recommended Medical Investigations in Pediatric Acute-Onset Neuropsychiatric Syndrome

**DOI:** 10.1001/jamanetworkopen.2026.2618

**Published:** 2026-03-20

**Authors:** Sara Vasiljevic, Malin E. Winerdal, Ronny Wickström, Eva Hesselmark, David Mataix-Cols, Max Winerdal, Malin Ek Sigerud, Selma Idring Nordström, Kristina Tedroff

**Affiliations:** 1 Centre for Psychiatry Research, Department of Clinical Neuroscience, Karolinska Institutet and Stockholm Health Care Services, Region Stockholm, Stockholm, Sweden; 2 Department of Laboratory Medicine, Karolinska Institutet, Stockholm, Sweden; 3 Clinical Immunology and Transfusion Medicine Unit, Karolinska University Hospital, Stockholm, Sweden; 4 Neuropediatric Unit, Department of Women’s and Children’s Health, Karolinska Institutet, Stockholm, Sweden; 5 Neuropediatric Unit, Astrid Lindgren Children’s Hospital, Karolinska University Hospital, Stockholm, Sweden; 6 Department of Clinical Sciences, Lund University, Lund, Sweden; 7 Division of Child and Adolescent Psychiatry, Uppsala Academic Hospital, Uppsala, Sweden; 8 Child and Adolescent Psychiatry Specialist Outpatient Care, Stockholm Health Care Services, Region Stockholm, Stockholm, Sweden

## Abstract

**Importance:**

The clinical utility of the comprehensive laboratory and medical investigations currently recommended for children with pediatric acute-onset neuropsychiatric syndrome (PANS) remains unclear. Notably, comparisons with relevant psychiatric control patients without suspected immunologic pathogenesis are lacking.

**Objective:**

To evaluate whether currently recommended laboratory investigations differentiate children with PANS from children with idiopathic obsessive-compulsive disorder (OCD) and/or tic disorders and whether the full set of recommended medical investigations identify underlying somatic conditions in children with PANS.

**Design, Setting, and Participants:**

This case-control study recruited children (aged 4-18 years) with PANS and children with idiopathic OCD and/or tic disorders (control group) from specialist PANS and OCD clinics in Stockholm, Sweden, between January 1, 2020, and September 19, 2023. The data analyses were performed between June 16, 2024, and August 19, 2025.

**Main Outcomes and Measures:**

Following published PANS guidelines, assessments included 56 laboratory variables from blood and throat cultures. Additional data from cerebrospinal fluid analysis, brain magnetic resonance imaging, and electroencephalography were available for a subsample of the PANS group. Laboratory findings were compared between the PANS and control groups.

**Results:**

Among 109 participants, the PANS group included 51 children (mean [SD] age, 10.2 [3.4] years; 34 boys [66.7%], and the control group included 58 children (mean [SD] age, 13.6 [3.1] years; 29 boys [50.0%]). Nearly all participants had at least 1 abnormal laboratory finding (44 [86.3%] in the PANS group, 56 [96.6%] in the control group), with no significant between-group differences. Most participants in the PANS group had 3 or more abnormal laboratory findings, while most in the control group had 4 or more. One participant with PANS was diagnosed with celiac disease; another showed electroencephalographic signs of neuroinflammatory activity without a definite diagnosis prior to the PANS assessment. Incidental, nonactionable findings were frequent.

**Conclusions and Relevance:**

In this case-control study of children with PANS and idiopathic OCD and/or tic disorders, abnormal laboratory findings were common in both groups and did not differ significantly. The full set of recommended medical investigations rarely identified underlying somatic conditions in children with PANS. These findings question the clinical utility of the comprehensive and costly medical investigations currently recommended for children with suspected PANS.

## Key Points

**Question:**

Does the comprehensive set of laboratory investigations recommended for children with pediatric acute-onset neuropsychiatric syndrome (PANS) differentiate them from children with idiopathic obsessive-compulsive disorder (OCD) and/or tic disorders, and do these investigations help identify underlying somatic conditions?

**Findings:**

In this case-control study of 51 children with PANS and a control group of 58 children with idiopathic OCD and/or tic disorders, nonspecific abnormal laboratory findings were common in both groups, with no between-group differences. The full set of medical investigations recommended for PANS failed to identify underlying somatic conditions in nearly all children with PANS.

**Meaning:**

These findings question the clinical utility of the comprehensive medical investigations currently recommended for suspected PANS.

## Teaser

This case-control study evaluates whether recommended laboratory tests for pediatric acute-onset neuropsychiatric syndrome (PANS) differentiate PANS from idiopathic obsessive-compulsive and tic disorders and identify underlying somatic conditions in children with PANS in Sweden.

## Introduction

Pediatric acute-onset neuropsychiatric syndrome (PANS), including pediatric autoimmune neuropsychiatric disorders associated with streptococcal infections (PANDAS),^1,2^ is defined by the sudden onset of obsessive-compulsive disorder (OCD), restricted food intake, and/or tics, along with at least 2 common psychiatric and/or neurologic symptoms.^1^ An immunologic etiology has been proposed,^1,2^ although solid evidence is lacking.^3^ Yet, this hypothesis has shaped the clinical management of PANS.

Clinical guidelines for suspected PANS recommend comprehensive medical investigations, including blood samples; throat swabs; and complementary cerebrospinal fluid (CSF) analysis, brain magnetic resonance imaging (MRI), and electroencephalography (EEG).^4,5^ These assessments aim to identify treatable components and underlying somatic conditions.^1^ Treatments for PANS include approaches that are otherwise not considered for idiopathic OCD, eating disorders, or tics, such as antibiotics and immunomodulatory interventions.^4,6,7^

To our knowledge, the recommended PANS investigations have never been systematically validated against other psychiatric conditions without suspected immunologic etiology. Previous studies have typically relied on retrospective designs and evaluated limited variables in small samples, sometimes including healthy control participants.^8^ Including idiopathic OCD and tic disorders as control conditions is particularly relevant because they share core clinical features with PANS yet are not hypothesized to be immunologically driven and are therefore managed using standard evidence-based psychiatric interventions. Moreover, somatic and autoimmune conditions have also been reported in individuals with idiopathic OCD and tic disorders,^9,10,11^ underscoring the need for comparison. The largest laboratory study on PANS, which included 193 individuals with symptom exacerbations,^12^ found nonspecific blood-based autoimmune markers in 54% and blood-based markers of immune dysregulation or inflammation in 12% but did not include a relevant control group for comparison.^12^ Similarly, conclusions from CSF, MRI, and particularly EEG studies in children with PANS have been limited by small sample sizes and inconclusive findings.^13,14,15,16,17,18^

Our study aimed to fill this important knowledge gap by assessing whether the recommended set of laboratory investigations differentiate children with PANS from a relevant psychiatric control group of children with idiopathic OCD and/or tic disorders. Additionally, we aimed to evaluate whether blood sampling and complementary CSF analyses, brain MRI, and EEG could help identify underlying somatic conditions, including immunologic disorders, in patients with PANS.

## Methods

### Study Design

This case-control study analyzed prospectively collected data from the longitudinal Karolinska PANS cohort of children with psychiatric symptoms of suspected immunologic etiology referred from pediatric or child and adolescent psychiatry clinics.^19^ The study adhered to the principles of the Declaration of Helsinki^20^ and was approved by the Swedish Ethical Review Committee. Families provided written informed consent for research use of clinical data. The study followed the Strengthening the Reporting of Observational Studies in Epidemiology (STROBE) reporting guideline.^21^

### Setting

Participants (aged 4-18 years) were consecutive referrals to 2 specialized clinics within the Child and Adolescent Psychiatry Research Center in Stockholm, Sweden. The immunopsychiatry clinic assesses and treats PANS and includes a multidisciplinary team of physicians specialized in child and adolescent psychiatry, pediatrics, and pediatric neurology; clinical psychologists; and registered psychiatric nurses. Regular consultations are also held with specialist pediatricians in neuroinflammation, infectious diseases, and rheumatology at Karolinska University Hospital to jointly assess and consider any underlying somatic conditions. The OCD and related disorders clinic delivers specialist care for children with these conditions, including tic disorders. Referrals initially sent to the immunopsychiatry clinic but diagnosed as idiopathic OCD or tic disorders are rerouted to this clinic for treatment.

### Participants

Between January 1, 2020, and September 19, 2023, consecutive patients at the immunopsychiatry clinic who met the following inclusion criteria were enrolled in the study: clinically significant OCD, restricted food intake and/or tic symptoms, informed consent, assessment according to guidelines, completed laboratory analyses within 2 months, and meeting strict PANS criteria, including PANDAS criteria (eFigure 1 in Supplement 1). Control participants met *Diagnostic and Statistical Manual of Mental Disorders* (Fifth Edition) criteria for OCD, tic disorder (ie, Tourette syndrome, chronic tic disorder, provisional tic disorder), or both. They were consecutively recruited from either the immunopsychiatry or OCD and related disorders clinics and comprehensively assessed by at least 1 physician and 1 clinical psychologist to exclude PANS. In an exploratory post hoc analysis, participants from these 2 clinics did not differ on any laboratory test results.

### Measurements

Suspected cases of PANS or PANDAS were evaluated per European clinical guidelines,^4^ which are closely aligned with North American guidelines.^5^ All assessments occurred at the same clinical research center to minimize misclassification bias and included comprehensive medical and psychiatric assessments by at least 1 physician and 1 clinical psychologist and a complete review of medical records; blood tests; throat swabs; and, when indicated, additional CSF analysis, brain MRI, and EEG. Multidisciplinary case conferences determined whether patients met PANS criteria.^1,2^

Laboratory data, including blood and throat cultures, collected within 2 months of the first visit and after symptom onset were included. Laboratory variables included multiple immune-related markers (eTable 1 in Supplement 1) and were evaluated against reference values by physicians from Karolinska University Hospital.^22^ All clinically relevant abnormal findings were further assessed by team physicians to consider underlying somatic conditions.

Neurologic symptoms prompted consultation with child neurologists for additional CSF analysis, brain MRI, and EEG. Complementary investigations within 6 months of the first visit were included and analyzed for immune activation, neuroinflammation, and other somatic conditions.

Data were collected using a standardized protocol and stored in an encrypted database. A random 15% sample was validated against patient records to confirm accuracy.

### Statistical Analysis

Data were analyzed between June 16, 2024, and August 19, 2025. Analyses were conducted using Stata/BE, version 18.0 (StataCorp LLC) and Statistica, version 14.0.1.25 (TIBCO Software Inc). Missing data were not imputed. For laboratory measurements outside assay detection limits, values below and above detection limits were imputed using half the lower limit and the maximum threshold, respectively.^23^

Laboratory differences between the PANS and control groups were analyzed using 2-sided Mann-Whitney *U* tests to account for extreme values, skewness, and unidirectional ranges. Dichotomous variables were analyzed using the χ^2^ test or Fisher exact test.

To reduce type I error, 31 of the 56 laboratory variables were selected a priori based on clinical relevance by specialists in pediatric neurology, immunology, and psychiatry (M.E.W., R.W., M.E.S., S.I.N., and K.T.) (eTable 1 in Supplement 1). The remaining variables are presented descriptively. Multiple testing was adjusted using the Benjamini-Hochberg correction, which offers a lower type II error risk than Bonferroni correction. A postcorrection threshold of *P* < .05 was considered statistically significant. Given small samples and nonnormal distributions, exact *P* values are reported.

Two sensitivity analyses were performed. First, to test the potential influence of delayed sampling, we examined whether time from symptom onset to laboratory testing (years) was associated with the total number of abnormal laboratory findings in the PANS group using negative binomial regression. Second, we explored whether covariation among laboratory variables could estimate group assignment (PANS or control) using principal component analysis (PCA) with the nonlinear iterative partial least squares algorithm,^24^ adjusting for sex and age. A 45% completeness threshold was applied to balance case and variable inclusion (n = 75-102). One control participant significantly skewed the model (identified via Hotelling *T*^2^) and was excluded.

## Results

### Demographic Characteristics

Among 109 participants, the PANS group included 51 children (including 5 meeting PANDAS criteria) (mean [SD] age, 10.2 [3.4] years; 34 boys [66.7%] and 17 girls [33.3%]), and the control group included 58 children (including 53 with OCD, 21 with a tic disorder, and 16 with OCD and a tic disorder) (mean [SD] age, 13.6 [3.1] years; 29 boys [50.0%] and 29 girls [50.0%]). The mean (SD) age at reported symptom onset was 8.1 (3.7) years for the PANS group and 10.6 (3.6) years for the control group. Mean (SD) duration from symptom onset to the first visit was 1.9 (2.1) years for the PANS group and 3.1 (3.0) years for the control group (Table 1).

**Table 1.** Demographic Characteristics in the PANS and Control Groups at the First Visit

Variable	Participants, No. (%)	
	PANS group (n = 51)	Control group (n = 58)
Age, mean (SD), y	10.2 (3.4)	13.6 (3.1)
Age at symptom onset, y		
Mean (SD)	8.1 (3.7)	10.6 (3.6)
Missing	6 (11.8)	5 (8.6)
Time from onset to assessment, y		
Mean (SD)	1.9 (2.1)	3.1 (3.0)
Missing	6 (11.8)	5 (8.6)
Sex		
Female	17 (33.3)	29 (50.0)
Male	34 (66.7)	29 (50.0)
CGAS^a^		
Mean (SD)	46 (13)	51 (13)
Missing	0	27 (46.6)
Clinical presentation		
Clinically significant OCD symptoms	45 (88.2)	50 (86.2)
Clinically significant tic symptoms	26 (51.0)	21 (36.2)
Comorbid autoimmune diagnosis^b^	1 (2.0)	6 (10.3)
Comorbid neurodevelopmental diagnosis^c^	12 (23.5)	18 (31.0)

Abbreviations: CGAS, Children’s Global Assessment Scale; *ICD-10*, *International Statistical Classification of Diseases, Tenth Revision*; OCD, obsessive-compulsive disorder; PANS, pediatric acute-onset neuropsychiatric syndrome.

^a^ Scored on a scale of 1 to 100, reflecting the lowest level of overall functioning in the past month; higher scores indicate higher functioning.

^b^ Autoimmune diagnoses included thyroid disease, celiac disease, psoriasis, type 1 diabetes mellitus, atopic eczema, and Henoch-Schönlein purpura, classified according to the *ICD-10*.

^c^ Neurodevelopmental diagnoses included attention-deficit/hyperactivity disorder, autism spectrum disorder, and intellectual disabilities, classified according to the *ICD-10* and/or the *Diagnostic and Statistical Manual of Mental Disorders* (Fifth Edition).

Preexisting *International Statistical Classification of Diseases, Tenth Revision* autoimmune conditions were found in 1 participant (2.0%) in the PANS group (Henoch-Schönlein purpura) and 6 participants (10.3%) in the control group (celiac disease, psoriasis, type 1 diabetes, thyroid disease, and atopic eczema). Preexisting *International Statistical Classification of Diseases, Tenth Revision* neurodevelopmental disorders (attention-deficit/hyperactivity disorder, autism, and intellectual disabilities) were registered in 12 participants (23.5%) with PANS and 18 control participants (31.0%) (Table 1).

### Laboratory Investigations

Among the 56 laboratory variables measured, abnormal findings of at least 1 were found in 44 participants (86.3%) in the PANS group and 56 (96.6%) in the control group. Most participants in the PANS group had 3 or more abnormal laboratory findings, while most control participants had 4 or more (Figure). For the 31 variables in the between-group analyses, the PANS group had a median of 27 analyzed variables (IQR, 19-28 variables), and the control group had a median of 28 variables (IQR, 27-31 variables), with 42 participants (82.4%) in the PANS group and 54 (93.1%) in the control group having at least 1 abnormal finding. The most common abnormal laboratory findings in the PANS group were low levels of ferritin (10 of 35 participants tested [28.6%]), low levels of leukocytes (12 of 48 participants tested [25.0%]), and low levels of complement component C4 (9 of 42 participants tested [21.4%]). In the control group, low levels of leukocytes (24 of 58 participants tested [41.4%]), low levels of orosomucoid (14 of 55 participants tested [25.5%]), and low levels of vitamin D (14 of 55 participants tested [25.5%]) were most frequent (eTable 2 in Supplement 1). Group A streptococcal infections were identified in 5 of 30 participants tested (16.7%) in the PANS group and 4 of 47 tested (8.5%) in the control group via throat culture. No statistically significant between-group differences were found for any laboratory variable (Table 2).

**Figure.**
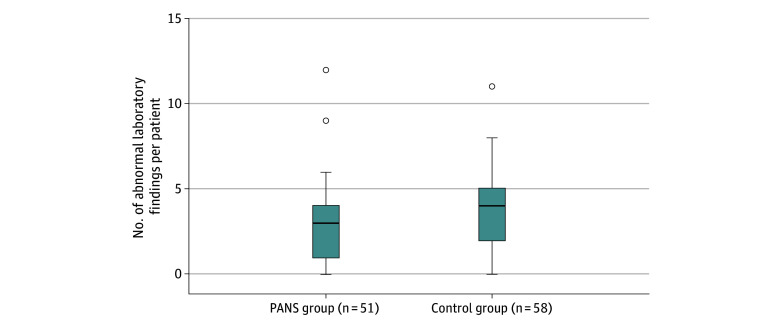
Box-and-Whisker Plots of Total Abnormal Laboratory Findings in the Pediatric Acute-Onset Neuropsychiatric Syndrome (PANS) Case and Control Groups The total number of abnormal laboratory findings per individual was aggregated across 56 immune-related variables. Boxes represent the IQR, horizontal lines within the box indicate the median, whiskers extend to the most extreme values within 1.5x IQR from the first and third quartiles, and points beyond the whiskers represent outliers.

**Table 2.** Statistical Comparisons of Laboratory Results Between the PANS and Control Groups^a^

Laboratory test^b^	Value, median (range)		*P* value	
	PANS group (n = 51)	Control group (n = 58)	BH corrected	Unadjusted
CBC with differential				
B-Hb, g/L	130.0 (110.0-153.0)	134.0 (109.0-157.0)	.62	.06
B-PLC, ×10^9^/L	304.0 (179.0-457.0)	273.5 (177.0-454.0)	.47	.12
B-WBC, ×10^9^/L	5.9 (2.9-16.1)	5.3 (3.2-15.0)	.46	.10
B-neutrophils, ×10^9^/L	2.6 (1.2-14.3)	2.5 (1.4-10.3)	.98	.98
B-lymphocytes, ×10^9^/L	2.4 (1.3-4.4)	1.9 (1.1-4.6)	.37	.01
B-eosinophils, ×10^9^/L	0.2 (0.1-1.4)	0.1 (0.1-0.9)	.20	.01
Inflammation, acute phase, protein factors				
S-CRP, mg/L	0.5 (0.5-4.0)	0.5 (0.5-50.0)	.82	.45
S-ESR, mm/h	5.0 (1.0-34.0)	4.0 (1.0-26.0)	.93	.75
S-α_1_-AT, g/L	1.2 (0.7-1.6)	1.2 (1.0-2.1)	.69	.27
S-orosomucoid, g/L	0.6 (0.3-1.3)	0.6 (0.3-1.7)	.81	.55
S-haptoglobin, g/L	0.6 (0.0-2.0)	0.6 (0.1-2.8)	.97	.91
S-AA, mg/L	1.1 (0.5-13.0)	1.7 (0.5-72.1)	.50	.16
S-ferritin, µg/L	26.0 (9.0-122.0)	30.0 (6.0-143.0)	.95	.83
P-cystatin-C, mg/L	0.9 (0.6-1.1)	0.9 (0.6-1.1)	.95	.80
S-albumin, g/L	41.0 (37.0-49.0)	41.0 (33.0-47.0)	.95	.86
Immunology				
S-IgA, g/L	1.0 (0.1-2.8)	1.3 (0.5-3.2)	.50	.08
S-IgM, g/L	1.0 (0.4-2.7)	1.0 (0.3-1.9)	.76	.46
S-IgG, g/L	9.8 (4.9-14.6)	10.1 (5.5-16)	.53	.15
Complement components, P-C1q, P-C3, P-C3d, P-C4				
≥1 Abnormal finding, No./total No. tested (%)	14/42 (33.3)	16/55 (29.1)	.90	.70
Cytokines				
S-IL-6, ng/L	1.0 (1.0-7.2)	1.0 (1.0-11.0)	.67	.28
S-IL-8, ng/L	6.0 (1.0-148.0)	6 (2.5-26.0)	.76	.49
S-IL-10, ng/L	2.5 (2.5-10.3)	2.5 (2.5-7.9)	.88	.65
S-IL-1β, ng/L	2.5 (2.5-4.0)	2.5 (2.5-33.9)	.53	.07
Autoantibodies				
S-ANA (IFL) positive, No./total No. (%)	3/45 (6.7)	0/55 (0.0)	.45	.09
S-anti-tTG, E/mL	0.5 (0.5-120.0)	0.5 (0.1-2.4)	.96	.93
S-anti-TPO, kE/L	5.0 (2.5-34.0)	5.0 (5.0-314.0)	.50	.18
Thyroid				
S-TSH, mE/L	1.8 (1.0-7.1)	2.2 (0.7-8.0)	.79	.46
S-T4, pmol/L	16.0 (10.0-21.0)	15.5 (10.0-29.0)	.86	.45
Other				
S-25(OH)D, nmol/L	63.0 (41.0-142.0)	60.0 (28.0-103.0)	.85	.60
P-ALT, µkat/L	0.3 (0.1-0.9)	0.3 (0.1-0.6)	.62	.28
Throat culture				
GAS bacteria positive, No./total No. tested (%)	5/30 (16.7)^c^	4/47 (8.5)	.62	.30

Abbreviations: 25(OH)D, 25-hydroxy vitamin D; α_1_-AT, α_1_-antitrypsin; AA, amyloid A; ALT, alanine aminotransferase; ANA, antinuclear antibody; B, blood; BH, Benjamini-Hochberg corrected; CBC, complete blood count; CRP, C-reactive protein; ESR, erythrocyte sedimentation rate; GAS, group A streptococcus; Hb, hemoglobin; IFL, immunofluorescence; Ig, immunoglobulin; IL, interleukin; P, plasma; PANS, pediatric acute-onset neuropsychiatric syndrome; PLC, platelet count; S, serum; T4, thyroxine; TPO, thyroid peroxidase; TSH, thyroid-stimulating hormone; tTG, tissue transglutaminase; WBC, white blood count.

^a^ Laboratory analyses conducted within 2 months of the first visit. To enable statistical comparisons, values below or above detection limits were imputed using either half the lower detection limit or the maximum measurable threshold. Statistical analyses were performed using 2-sided Mann-Whitney *U* tests for continuous or ordinal variables. One dichotomous variable (ANA positivity) was analyzed using the χ^2^ test, and the remaining 2 dichotomous variables (complement component abnormality and GAS positivity) were analyzed using Fisher exact tests due to low expected cell counts.

^b^ Conversion factors: Hb to g/dL, divide by 10; PLC to 10^3^/µL, divide by 1; WBC, neutrophils, lymphocytes, and eosinophils to /µL, divide by 0.001; CRP to mg/dL, divide by 10; α_1_-AT to µmol/L, multiply by 100 to convert to mg/dL, then multiply by 18.4; haptoglobin to mg/dL, divide by 100; ferritin to ng/mL, divide by 1; albumin to g/dL, divide by 10; IgA, IgM, and IgG to mg/dL, divide by 10; ALT to U/L, divide by 0.0167.

^c^ Of whom 1 participant was diagnosed with group A or group G streptococcal bacteria.

### Sensitivity Analyses

#### Influence of Delayed Sampling in the PANS Group

Significant overdispersion in the data (*P* = .001) necessitated negative binomial regression analysis for 48 participants. Shorter time from symptom onset to laboratory testing was associated with more abnormal findings (β [SE], −0.15 [0.06]; *z* = −2.49; *P* = .01). However, the model explained less than 3% of variance (pseudo-*R*^2^ = 0.0295). Four outliers with at least 9 abnormal findings influenced the result, including 1 participant in the PANS group identified with neuroinflammatory activity but no clear diagnosis; 1 with a preexisting PANDAS diagnosis; and 2 with PANS, including 1 with active group A streptococcal infection (eFigure 2 in Supplement 1).

#### Covariation of Laboratory Variables

The PCA identified 2 uncorrelated components (eFigure 3 in Supplement 1) selected via scree plot, explaining 20.3% of the variance. Principal component 1 reflected high loadings of inflammatory markers, including erythrocyte sedimentation rate and haptoglobin, with differences between the PANS and control groups (median [IQR], 0.04 [−1.28 to 1.62] vs 0.29 [−0.88 to 2.30], respectively; *P* = .23). Principal component 2 was associated with age at initial visit and at symptom onset, with significant differences between the PANS and control groups (median [IQR], −1.13 [−2.26 to 0.35] vs 0.57 [−0.76 to 2.30], n = 47 and 55, respectively; *P* < .001). Age at initial visit explained 67.0% (*r*^2^ = 0.67; *P* < .001) of the principal component 2 variance, confirming age rather than group assignment (PANS or control) as the main factor associated with the variance.

### Complementary CSF Analyses, Brain MRI, and EEG in the PANS Group

A total of 18 participants (35.3%) in the PANS group underwent complementary investigations (Table 3). Among the participants undergoing CSF analysis, 0 had oligoclonal bands, 1 of 14 tested (7.1%) had pleocytosis, and 2 of 9 tested (22.2%) had slightly elevated albumin levels (279 and 277 g/L; reference <260 g/L [to convert to g/dL, divide by 10]). Brain MRI was performed in 12 participants, with no signs of neuroinflammation and 5 (41.7%) showing benign cysts. Fifteen participants underwent EEG, including 4 (26.7%) undergoing additional sleep EEGs. Three EEG assessments showed abnormal results. Two showed slight intermittent focal slowing, with 1 assessed as a normal variant and the other showing normalized results on a follow-up EEG. The third EEG assessment showed persistent generalized slowing for 6 weeks with additional pleocytosis in CSF.

**Table 3.** Complementary CSF Analytic, Brain MRI, and EEG Findings in the PANS Group (n = 18)^a^

Test	Participants, No./total No. tested (%)
CSF analysis	
Pleocytosis	1/14 (7.1)
Increased levels of protein fraction, including albumin	2/9 (22.2)
Oligoclonal bands	0/14
Brain MRI	
Incidental findings	5/12 (41.7)
Signs of neuroinflammation	0/12
Routine EEG analysis	
Additional sleep-deprived EEG	4/15 (26.7)
Generalized slowing^b^	1/15 (6.7)
Focal abnormality	2/15 (13.3)^c^
Epileptiform activity	0/15

Abbreviations: CSF, cerebrospinal fluid; EEG, electroencephalography; MRI, magnetic resonance imaging; PANS, pediatric acute-onset neuropsychiatric syndrome.

^a^ Cerebrospinal fluid analysis, brain MRI, and EEG performed within 6 months of the first visit.

^b^ As reported by neurophysiologists.

^c^ Of whom 1 participant was classified as having a normal variant and the other showing normalization of findings on follow-up EEG.

### Somatic Conditions Identified in Participants With PANS

Celiac disease was identified in 1 participant with PANS through transglutaminase antibodies in the blood. Another case participant initially assessed for autoimmune encephalitis at a pediatric neurology clinic had 9 abnormal laboratory findings and abnormal EEG results compatible with encephalitis prior to the PANS referral and assessment, and despite comprehensive neurologic and immunologic workup at the pediatric neurology clinic, no clear diagnosis was established.

## Discussion

To our knowledge, this case-control study is the first to evaluate whether the comprehensive laboratory investigations recommended in European and North American PANS guidelines differentiate children with PANS from children with idiopathic OCD and/or tic disorders.^4,5^ The latter is an ideal control group because although much of the clinical presentation overlaps with PANS, idiopathic OCD and tic disorders have not been associated with immune-mediated mechanisms and are consequently managed using conventional psychiatric approaches. We also investigated whether the full set of recommended medical investigations was helpful in identifying underlying somatic conditions in children with PANS. Abnormal laboratory findings were observed in nearly all participants in both groups, with no significant differences across different statistical approaches. Celiac disease was identified in 1 participant with PANS through blood analyses. Additional investigations, including CSF analysis, brain MRI, and EEG, in the PANS group failed to identify underlying somatic conditions in nearly all participants with PANS. Only 1 participant showed signs of neuroinflammatory activity prior to the PANS assessment, though no clear neuroinflammatory diagnosis was established. These findings question the clinical utility of the comprehensive medical investigations currently recommended in PANS guidelines.

Nearly all participants with PANS (44 of 51 [86.3%]) and control participants (56 of 58 [96.6%]) exhibited at least 1 nonspecific abnormal laboratory finding across the full set of 56 variables (42 [82.4%] and 54 [93.1%] across the 31 variables in the between-group analyses). In fact, most participants in the control group had slightly more abnormal findings (≥4) than those in the PANS group (≥3).

Compared with Stanford PANS cohort, the largest laboratory study on PANS to date,^12^ our study found higher rates of immunologic abnormal findings, such as low levels of leukocytes, but lower rates of nonspecific autoimmune abnormal findings, including antinuclear antibody positivity.^12^ Of note, the Karolinska and Stanford PANS cohorts were demographically similar. Our cohort included slightly more boys (66.7% vs 58.0%), with comparable mean (SD) ages at symptom onset (8.1 [3.4] vs 7.5 [3.5] years) and at first clinical visit (10.2 [3.4] vs 9.8 [3.9] years). Notably, our control participants, who were older than the participants in our PANS group, had similar rates of autoimmune disorders to those reported in the longitudinal follow-up of the Stanford PANS cohort,^12^ including celiac disease. Consistent with our findings, previous studies have reported a wide range of nonspecific abnormal laboratory findings in patients with PANS,^3^ including calcium/calmodulin-dependent protein kinase II activity and dopamine receptor autoantibodies, but these have failed to reveal diagnostic value even compared with healthy control patients.^25^ Interestingly, an older study from the Stanford PANS cohort found no significant differences in immunologic markers, such as erythrocyte sedimentation rate and antinuclear antibody positivity, between strict PANS and nonacute PANS.^26^

In line with previous reports from the Stanford^12^ and Karolinska^27^ PANS cohorts, abnormal complement levels, most commonly C4 and C3, were frequently observed in both groups (eTable 2 in Supplement 1). However, complement values should be interpreted collectively and are prone to false–weakly positive test results due to complement activation in the test tube ex vivo.^28^ Moreover, age-adjusted reference ranges are not generally applied for complement values in Sweden, despite reported age-related differences in complement concentrations.^29^ In our sample, 17 of 25 C4 values outside the standard reference range (68.0%) were within suggested age-adjusted norms,^29^ and 5 of 8 (62.5%) of the remaining cases were from the control group. Although some participants had low C3 concentrations, elevated C3d, indicating complement activation or C3 consumption, was rare (n = 3 per group).

Time between symptom onset and workup is relevant as many immunologic markers, such as C-reactive protein and haptoglobin, are only present in proximity to an immunologic event. However, all case participants exhibited PANS symptoms at assessment, and similar delays between symptom onset and laboratory investigations have often been reported in PANS cohorts.^12,27,30,31^ Sensitivity analysis in our PANS group showed a negative association between time since onset and the number of abnormal laboratory findings, which explained less than 3% of the variance and was driven by 4 outliers (eFigure 2 in Supplement 1). Moreover, PCA identified age, not inflammatory markers, as a significant component differentiating the PANS group from the control group.

The full set of recommended medical investigations, including complementary CSF analysis, brain MRI, and EEG, failed to identify relevant underlying somatic conditions, such as neuroinflammation, in nearly all participants with PANS. One case of celiac disease was identified through laboratory results and another showed abnormal laboratory and EEG findings suggestive of encephalitis, but comprehensive neurologic investigations did not yield a clear diagnosis, prompting referral of the patient to our clinic. Moreover, incidental, nonactionable findings were common. These findings align with the limited and inconsistent literature on CSF, MRI, and EEG analyses in PANS. A recent systematic review on CSF suggested shared mechanisms between PANDAS and Sydenham chorea while underscoring the scarcity of studies.^13^ In the largest CSF study in PANS (N = 35), elevated CSF protein and/or albumin quotient was reported in approximately one-fourth of participants.^32^ Magnetic resonance imaging studies have suggested volume changes in brain regions of individuals with PANDAS^14^ and cerebral microstructural differences in patients with PANS,^16^ but results remained inconsistent and samples were small.^14,16,17^ Similarly, EEG studies in PANS are limited, with 1 reporting focal epileptiform abnormalities in a small subset of patients^17^ and another finding normal EEG results prior to treatment.^18^ Similar findings from CSF,^33^ MRI,^34^ and EEG^35^ analyses have been reported in children with neurodevelopmental disorders, which were present in nearly one-fourth of our PANS cohort. Collectively, these findings question the value of routinely ordering such investigations due to their potentially invasive nature and cost.

### Limitations

This study had some limitations. There was a mean 1.9-year delay from symptom onset to assessment in the PANS group, as commonly reported in the PANS literature.^12,27,30,31^ Sensitivity analyses indicated a minimal influence of this delay on laboratory results, and all case participants had ongoing PANS symptoms at assessment. The absence of control data for CSF analyses, brain MRI, and EEG restricted comparisons. Selection bias is a potential concern, as individuals presenting with immunologic markers may be more likely to receive a PANS diagnosis. Such bias would typically suggest more abnormal findings in the PANS group, which were not observed. Interpretation is limited by sample size. However, neither descriptive statistics nor PCA revealed clear differences between the PANS and control groups. In fact, a marginally higher rate of abnormal laboratory findings was observed among control participants, suggesting that any possible differences between groups were small and of uncertain clinical utility. Larger, well-powered prospective studies are needed to detect potentially small associations.

## Conclusions

This case-control study of children with PANS and idiopathic OCD and/or tic disorders found no significant differences between groups across laboratory variables. Furthermore, the full set of recommended medical investigations, including complementary CSF analyses, brain MRI, and EEG, rarely identified underlying somatic conditions in PANS and often produced incidental, nonactionable findings. Given the potentially invasive nature and cost of these investigations, we urge clinicians to make judicious decisions regarding the implementation of the current recommendations.

## Notes

Supplement 1. **eFigure 1.** Flowchart of Data Collection for the Karolinska PANS Cohort and Control Group **eTable 1.** Summary of Variables on Blood and Throat Culture by Category **eTable 2.** Laboratory Findings in the PANS and Control Groups Across 56 Variables **eFigure 2.** Association Between Time From Symptom Onset to Laboratory Testing and Number of Laboratory Abnormalities in the PANS Group **eFigure 3. **Principal Component Analysis of Laboratory Variables With Age and Sex as Unbiased Covariates

Supplement 2.
Data Sharing Statement
