# Supplementary material for: Recommended Medical Investigations in Pediatric Acute-Onset Neuropsychiatric Syndrome
Source: JAMA Netw Open. 2026 Mar 20;9(3):e262618. doi: 10.1001/jamanetworkopen.2026.2618 (PMC13005155; doi:10.1001/jamanetworkopen.2026.2618)
Supplement: Supplement 1. — eFigure 1. Flowchart of Data Collection for the Karolinska PANS Cohort and Control Group eTable 1. Summary of Variables on Blood and Throat Culture by Category eTable 2. Laboratory Findings in the PANS and Control Groups Across 56 Variables eFigure 2. Association Between Time From Symptom Onset to Laboratory Testing and Number of Laboratory Abnormalities in the PANS Group eFigure 3. Principal Component Analysis of Laboratory Variables With Age and Sex as Unbiased Covariates [file jamanetwopen-e262618-s001.pdf]

Supplemental Online Content

Vasiljevic S, Winerdal ME, Wickström R, et al. Reconsidering recommended medical investigations in pediatric acute-onset neuropsychiatric syndrome. *JAMA Netw Open*. 2026;9(3):e262618. doi:10.1001/jamanetworkopen.2026.2618

eFigure 1. Flowchart of Data Collection for the Karolinska PANS Cohort and Control Group

eTable 1. Summary of Variables on Blood and Throat Culture by Category

eTable 2. Laboratory Findings in the PANS and Control Groups Across 56 Variables

eFigure 2. Association Between Time From Symptom Onset to Laboratory Testing and Number of Laboratory Abnormalities in the PANS Group

eFigure 3. Principal Component Analysis of Laboratory Variables With Age and Sex as Unbiased Covariates

This supplemental material has been provided by the authors to give readers additional information about their work.

© 2026 Vasiljevic S et al. *JAMA Network Open*.

eFigure 1. Flowchart of Data Collection for the Karolinska PANS Cohort and Control Group

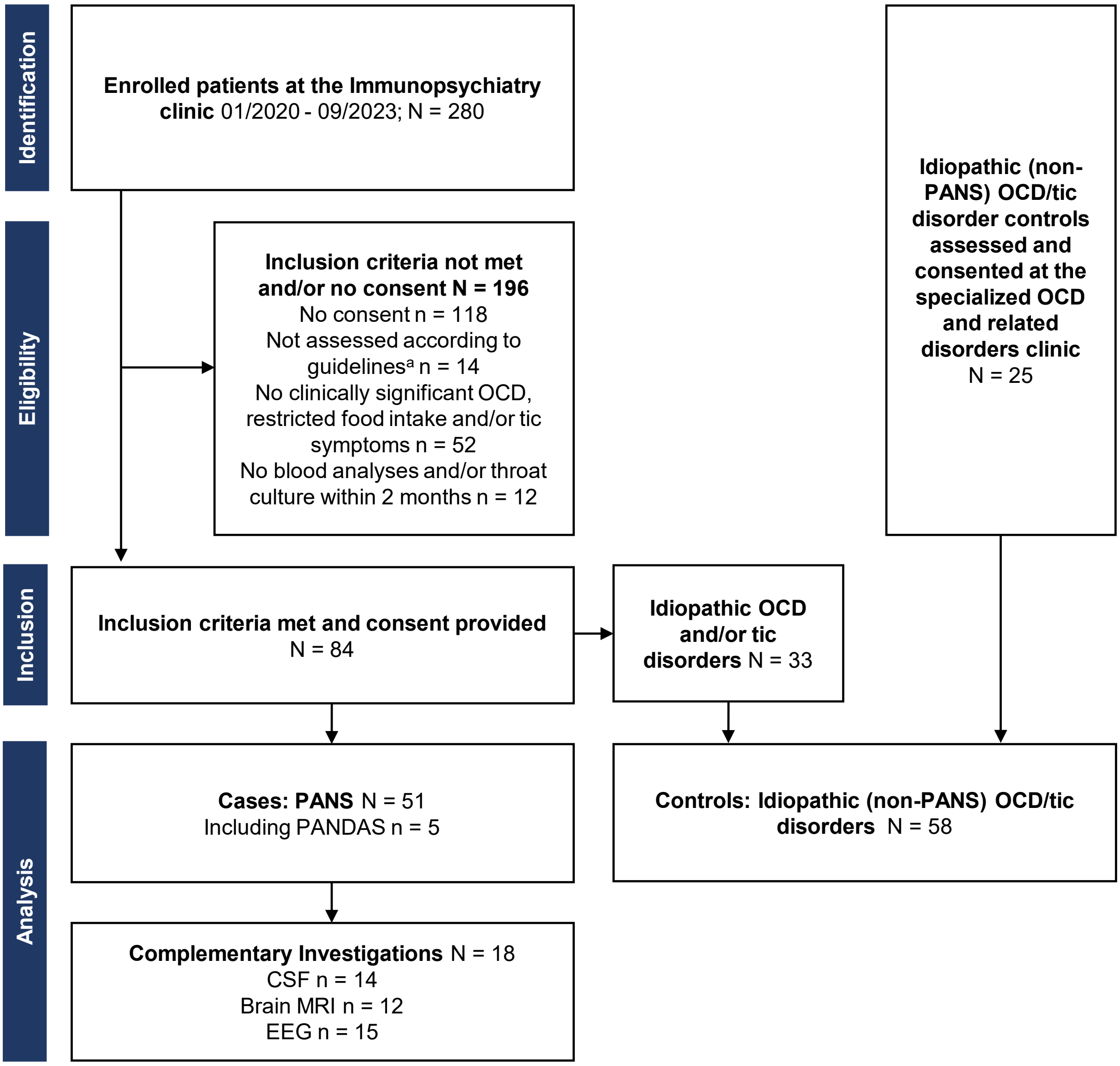

[a]Including cases referred for consultative assessment, e.g., patients residing in other counties.
Abbreviations: **OCD**, obsessive-compulsive disorder; **PANS**, Pediatric Acute-Onset Neuropsychiatric Syndrome; **PANDAS**, Pediatric Autoimmune Neuropsychiatric Disorder Associated with Streptococcal Infection; **CSF**, cerebrospinal fluid; **brain MRI**, brain magnetic resonance imaging; **EEG**, electroencephalogram.

© 2026 Vasiljevic S et al. *JAMA Network Open*.

eTable 1. Summary of Variables on Blood and Throat Culture by Category

Category	Variable, abbreviation (unit)
**Complete Blood Count (CBC) with differential (B= blood)**	Hemoglobin, B-Hb (g/L)
	Platelets, B-PLC (x10(9)/L)
	White Blood Cell Count, B-WBC (x10(9)/L)
	B-Neutrophils (x10(9)/L)
	B-Lymphocytes (x10(9)/L)
	B-Eosinophils (x10(9)/L)
	B-Basophils[a] (x10(9)/L)
	B-Monocytes[a] (x10(9)/L)
	Red blood cell count[a], B-RBC (x10(12)/L)
	Erythrocyte Volume Fraction[a], B-EVF (volume of red blood cells/total volume of blood)
	Erythrocytes Mean Corpuscular Volume[a], Erc B-MCV (fL)
	Erythrocytes Mean Corpuscular Hemoglobin[a], Erc B-MCH (pg)
**Inflammation/Acute phase/Protein factors (S= Serum; P= Plasma)**	C-Reactive Protein, S-CRP (mg/L)
	Erythrocyte Sedimentation Rate, S-ESR (mm)
	Alpha-1-Antitrypsin, S-α1AT (g/L)
	Orosomucoid, S-Orosomucoid (g/L)
	Haptoglobin, S-Hp (g/L)
	Amyloid A, S-AA (mg/L)
	Ferritin, S-Ferritin (microg/L)
	Cystatin-C, P-Cystatin-C (mg/L)
	S-Albumin (g/L)
**Immunology (S= Serum)**	Immunoglobulin A, S-IgA (g/L)
	Immunoglobulin M, S-IgM (g/L)
	Immunoglobulin G, S-IgG (g/L)
	Immunoglobulin G1[a], S-IgG1 (g/L)
	Immunoglobulin G2[a], S-IgG2 (g/L)
	Immunoglobulin G3[a], S-IgG3 (g/L)
	Immunoglobulin G4[a], S-IgG4 (g/L)
**Complement components (P= Plasma)**	≥1 complement component abnormality (yes/no)
	Complement component 1q[a], P-C1q (mg/L)
	Complement component 3[a], P-C3 (g/L)
	Complement component 3d[a], P-C3d (mg/L)
	Complement component 4[a], P-C4 (g/L)
**Cytokines (S= Serum)**	Interleukin-6, S-IL-6 (ng/L)
	Interleukin-8, S-IL-8 (ng/L)
	Interleukin-10, S-IL-10 (ng/L)
	Interleukin-1β, S-IL-1β (ng/L)
**Autoantibodies (S=Serum)**	Anti-Nuclear antibodies, S-ANA (IFL), (positive/negative)
	Anti-Tissue Transglutaminase-antibodies, S-anti-tTG (E/mL)
	Anti-Thyroid Peroxidase antibodies, S-anti-TPO (kE/L)
	Anti-Nucleosome antibodies[a], S-anti-NCS (E/mL)
	Anti-Ribosomal P protein antibodies, S-anti-Rib-P[a] (E/mL)
	Anti-Ribonucleoprotein 68 antibodies, S-anti-RNP68[a] (E/mL)
	Anti-Topoisomerase I antibodies, S-anti-Scl-70[a] (E/mL)
	Anti-Smith antibodies, S-anti-Sm[a] (E/mL)
	Anti-Smith and ribonucleoprotein antibodies, S-anti-SmRNP[a] (E/mL)
	Anti-Sjögren's Syndrome A antibodies (Ro52), S-anti-SS-A[a] (Ro52) (E/mL)
	Anti-Sjögren's Syndrome A antibodies (Ro60), S-anti-SS-A[a] (Ro60) (E/mL)

© 2026 Vasiljevic S et al. *JAMA Network Open*.

	Anti-Sjögren's Syndrome B antibodies, S-anti-SS-B[a] (E/mL)
	Anti-Centromere protein B antibodies, S-anti-CENPB[a] (E/mL)
	Anti-Histidyl tRNA synthetase antibodies, S-anti-Jo-1[a] (E/mL)
	Anti-double stranded DNA antibodies, S-anti-dsDNA[a] (IE/mL)
**Thyroid (S=Serum)**	Thyroid Stimulating Hormone, S-TSH (mE/L)
	Thyroxine, S-T4 (pmol/L)
**Other (S=Serum, P= Plasma)**	25-Hydroxyvitamin D, S-25(OH)D (nmol/L)
	Alanine Aminotransferase, P-ALT (mikrokat/L)
**Throat culture**	Group A streptococci, GAS (positive/negative)

[a]Variables presented descriptively/not included in the statistical between-group analysis

© 2026 Vasiljevic S et al. *JAMA Network Open*.

eTable 2. Laboratory Findings in PANS and Control Groups Across 56 Variables

**Complete Blood Count (CBC) with differential**	**PANS** (N = 51)	**Controls** (N = 58)
**B-Hb** g/L, reference 105–150		
Below reference, No./total No. (%)	1/47 (2.1)	0/58 (0.0)
Above reference, No./total No. (%)	0/47 (0.0)	0/58 (0.0)
Median (range)	130.0 (110.0-153.0)	134.0 (109.0-157.0)
**B-PLC** x10(9)/L, reference 150-400		
Below reference, No./total No. (%)	0/47 (0.0)	0/58 (0.0)
Above reference, No./total No. (%)	4/47 (8.5)	6/58 (10.3)
Median (range)	304.0 (179.0-457.0)	273.5 (177.0-454.0)
**B-WBC** x10(9)/L, reference 5.0-13.0		
Below reference, No./total No. (%)	12/48 (25.0)	24/58 (41.4)
Above reference, No./total No. (%)	1/48 (2.1)	1/58 (1.7)
Median (range)	5.9 (2.9-16.1)	5.3 (3.2-15.0)
**B-Neutrophils** x10(9)/L, reference 2.8-8.4		
Below reference, No./total No. (%)	7/42 (16.7)	13/56 (23.2)
Above reference, No./total No. (%)	1/42 (2.4)	1/56 (1.8)
Median (range)	2.6 (1.2-14.3)	2.5 (1.4-10.3)
**B-Lymphocytes** x10(9)/L, reference 1.5-6.5		
Below reference, No./total No. (%)	2/42 (4.8)	1/56 (1.8)
Above reference, No./total No. (%)	0/42 (0.0)	0/56 (0.0)
Median (range)	2.4 (1.3-4.4)	1.9 (1.1-4.6)
**B-Eosinophils** x10(9)/L, reference 0.0-0.5		
Above reference, No./total No. (%)	3/42 (7.1)	1/56 (1.8)
Median (range)	0.2 (0.1-1.4)	0.1 (0.1-0.9)
**B-Basophils** x10(9)/L, reference <0.1		
Above reference, No./total No. (%)	0/42 (0.0)	0/56 (0.0)
Median (range)	0.1 (0.0-0.5)	0.1 (0.0-0.1)
**B-Monocytes** x10(9)/L, reference 0.2-0.8		
Below reference, No./total No. (%)	1/42 (2.4)	0/56 (0.0)
Above reference, No./total No. (%)	2/42 (4.8)	2/56 (3.6)
Median (range)	0.4 (0.1-1.8)	0.4 (0.2-1.1)
**B-RBC** x10(12)/L, reference 4.1–5.3		
Below reference, No./total No. (%)	3/47 (6.4)	5/58 (8.6)
Above reference, No./total No. (%)	2/47 (4.3)	2/58 (3.4)
Median (range)	4.6 (3.9-5.5)	4.7 (3.7-5.7)
**B-EVF** L/L, reference 0.37-0.41		
Below reference, No./total No. (%)	7/47 (14.9)	13/58 (22.4)
Above reference, No./total No. (%)	0/47 (0.0)	2/58 (3.4)
Median (range)	0.4 (0.3-0.5)	0.4 (0.3-0.5)
**Erc B-MCV** fL, reference 76-94		
Below reference, No./total No. (%)	1/47 (2.1)	1/58 (1.7)
Above reference, No./total No. (%)	0/47 (0.0)	0/58 (0.0)
Median (range)	83.0 (75.0-94.0)	86.0 (65.0-92.0)
**Erc B-MCH** pg, reference 26-30		
Below reference, No./total No. (%)	4/47 (8.5)	5/58 (8.6)
Above reference, No./total No. (%)	2/47 (4.3)	2/58 (3.4)
Median (range)	28.0 (25.0-32.0)	29.0 (20.0-32.0)
**Inflammation/Acute phase/Protein factors**	**PANS** (N = 51)	**Controls** (N = 58)
**S-CRP** mg/L, reference <3.0		
Above reference, No./total No. (%)	2/43 (4.7)	4/56 (7.1)
Median (range)	0.5 (0.5-4.0)	0.5 (0.5-50.0)
**S-ESR** mm, reference <20		
Above reference, No./total No. (%)	5/45 (11.1)	4/54 (7.4)
Median (range)	5.0 (1.0-34.0)	4.0 (1.0-26.0)
**S-α1AT** g/L, reference 1.1-1.8		
Below reference, No./total No. (%)	5/38 (13.2)	4/55 (7.3)

© 2026 Vasiljevic S et al. *JAMA Network Open*.

Inflammation/Acute phase/Protein factors	**PANS** (N = 51)	**Controls** (N = 58)
**S-α1AT** g/L, reference 1.1-1.8		
Above reference, No./total No. (%)	0/38 (0.0)	2/55 (3.6)
Median (range)	1.2 (0.7-1.6)	1.2 (1.0-2.1)
**S-Orosomucoid** g/L, reference 0.4-1.1		
Below reference, No./total No. (%)	5/38 (13.2)	14/55 (25.5)
Above reference, No./total No. (%)	0/38 (0.0)	1/55 (1.8)
Median (range)	0.6 (0.3-1.3)	0.6 (0.3-1.7)
**S-Haptoglobin** g/L, reference <1.6		
Above reference, No./total No. (%)	1/38 (2.6)	1/55 (1.8)
Median (range)	0.6 (0.0-2.0)	0.6 (0.1-2.8)
**S-AA** mg/L, reference <10.0		
Above reference, No./total No. (%)	1/36 (2.8)	2/55 (3.6)
Median (range)	1.1 (0.5-13.0)	1.7 (0.5-72.1)
**S-Ferritin** microg/L, reference 30-400		
Below reference, No./total No. (%)	10/35 (28.6)	10/47 (21.3)
Above reference, No./total No. (%)	0/35 (0.0)	1/47 (2.1)
Median (range)	26.0 (9.0-122.0)	30.0 (6.0-143.0)
**P-Cystatin-C** mg/L, reference 0.6-1.1		
Below reference, No./total No. (%)	0/37 (0.0)	1/54 (1.9)
Above reference, No./total No. (%)	0/37 (0.0)	0/54 (0.0)
Median (range)	0.9 (0.6-1.1)	0.9 (0.6-1.1)
**S-Albumin** g/L, reference 37-47		
Below reference, No./total No. (%)	0/39 (0.0)	4/54 (7.4)
Above reference, No./total No. (%)	1/39 (2.6)	0/54 (0.0)
Median (range)	41.0 (37.0-49.0)	41.0 (33.0-47.0)
**Immunology**	**PANS** (N = 51)	**Controls** (N = 58)
**S-IgA** g/L, reference 0.50-2.20		
Below reference, No./total No. (%)	3/41 (7.3)	0/54 (0.0)
Above reference, No./total No. (%)	1/41 (2.4)	1/54 (1.9)
Median (range)	1.0 (0.1-2.8)	1.3 (0.5-3.2)
**S-IgM** g/L, reference 0.50-1.7		
Below reference, No./total No. (%)	1/41 (2.4)	4/54 (7.4)
Above reference, No./total No. (%)	3/41 (7.3)	1/54 (1.9)
Median (range)	1.0 (0.4-2.7)	1.0 (0.3-1.9)
**S-IgG** g/L, reference 6.60-15.30		
Below reference, No./total No. (%)	2/41 (4.9)	1/54 (1.9)
Above reference, No./total No. (%)	1/41 (2.4)	1/54 (1.9)
Median (range)	9.8 (4.9-14.6)	10.1 (5.5-16.0)
**S-IgG1*** g/L, reference 3.70-9.30		
Below reference, No./total No. (%)	1/34 (2.9)	2/56 (3.6)
Above reference, No./total No. (%)	1/34 (2.9)	5/56 (8.9)
Median (range)	6.1 (2.5-10.1)	5.8 (2.6-9.6)
**S-IgG2** g/L, reference 1.00-4.00		
Below reference, No./total No. (%)	0/34 (0.0)	0/56 (0.0)
Above reference, No./total No. (%)	2/34 (5.9)	2/56 (3.6)
Median (range)	2.4 (0.5-5.1)	2.7 (1.3-6.1)
**S-IgG3** g/L, reference 0.22-1.09		
Below reference, No./total No. (%)	3/34 (8.8)	4/56 (7.1)
Above reference, No./total No. (%)	4/34 (11.8)	4/56 (7.1)
Median (range)	0.6 (0.2-1.8)	0.6 (0.1-1.9)
**S-IgG4** g/L, reference 0.04-1.90		
Below reference, No./total No. (%)	0/34 (0.0)	1/56 (1.8)
Above reference, No./total No. (%)	1/34 (2.9)	1/56 (1.8)
Median (range)	0.4 (0.1-10.1)	0.4 (0.0-2.6)
**Complement components**	**PANS** (N = 51)	**Controls** (N = 58)

© 2026 Vasiljevic S et al. *JAMA Network Open*.

**Complement components**	**PANS** (N = 51)	**Controls** (N = 58)
**P-C1q** mg/L, reference 176-312		
Below reference, No./total No. (%)	0/42 (0.0)	3/55 (5.5)
Above reference, No./total No. (%)	1/42 (2.4)	0/55 (0.0)
Median (range)	112.0 (72.0-323.0)	122.0 (70.0-254.0)
**P-C3** g/L, reference 0.76-1.52		
Below reference, No./total No. (%)	3/42 (7.1)	7/55 (12.7)
Above reference, No./total No. (%)	0/42 (0.0)	0/55 (0.0)
Median (range)	1.0 (0.6-1.4)	1.0 (0.5-1.5)
**P-C3d** mg/L, reference <8		
Above reference, No./total No. (%)	3/42 (7.1)	3/54 (5.6)
Median (range)	5.5 (2.0-18.0)	5.0 (2.0-10.0)
**P-C4** g/L, reference 0.13-0.32		
Below reference, No./total No. (%)	9/42 (21.4)	12/55 (21.8)
Above reference, No./total No. (%)	3/42 (7.1)	1/55 (1.8)
Median (range)	0.2 (0.1-0.4)	0.2 (0.1-0.5)
**Cytokines**	**PANS** (N = 51)	**Controls** (N = 58)
**S-IL-6** ng/L, reference <7.0		
Above reference, No./total No. (%)	1/37 (2.7)	1/52 (1.9)
Median (range)	1.0 (1.0-7.2)	1.0 (1.0-11.0)
**S-IL-8** ng/L, reference <60.0		
Above reference, No./total No. (%)	2/15 (13.3)	0/26 (0.0)
Median (range)	6.0 (1.0-148.0)	6.0 (2.5-26.0)
**S-IL-10** ng/L, reference <5.0		
Above reference, No./total No. (%)	1/12 (8.3)	1/26 (3.8)
Median (range)	2.5 (2.5-10.3)	2.5 (2.5-7.9)
**S-IL-1β** ng/L, reference <5.0		
Above reference, No./total No. (%)	0/13 (0.0)	4/26 (15.4)
Median (range)	2.5 (2.5-4.0)	2.5 (2.5-33.9)
**Autoantibodies**	**PANS** (N = 51)	**Controls** (N = 58)
**S-ANA (IFL)** positive/negative		
Positive, No./total No. (%)	3/45 (6.7)	0/55 (0.0)
**S-anti-tTG** E/mL, reference <7.0		
Above reference, No./total No. (%)	1/43 (2.3)	0/53 (0.0)
Median (range)	0.5 (0.5-120.0)	0.5 (0.1-2.4)
**S-anti-TPO** kE/L, reference <34		
Above reference, No./total No. (%)	0/41 (0.0)	3/54 (5.6)
Median (range)	5.0 (2.5-34.0)	5.0 (5.0-314.0)
**S-anti-Nucleosome** E/mL, reference <1.0		
Above reference, No./total No. (%)	0/44 (0.0)	0/55 (0.0)
Median (range)	0.5 (0.5-0.5)	0.5 (0.5-0.5)
**S-anti-Rib-P** E/mL, reference <1.0		
Above reference, No./total No. (%)	1/44 (2.3)	1/55 (1.8)
Median (range)	0.5 (0.5-1.1)	0.5 (0.5-1.3)
**S-anti-RNP68** E/mL, reference <1.0		
Above reference, No./total No. (%)	0/44 (0.0)	0/55 (0.0)
Median (range)	0.5 (0.5-0.5)	0.5 (0.5-0.5)
**S-anti-Scl-70** E/mL, reference <1.0		
Above reference, No./total No. (%)	0/44 (0.0)	0/55 (0.0)
Median (range)	0.5 (0.5-0.5)	0.5 (0.5-0.5)
**S-anti-Sm** E/mL, reference <1.0		
Above reference, No./total No. (%)	0/44 (0.0)	0/55 (0.0)
Median (range)	0.5 (0.5-0.5)	0.5 (0.5-0.5)
**S-anti-SmRNP** E/mL, reference <1.0		
Above reference, No./total No. (%)	0/44 (0.0)	1/55 (1.8)
Median (range)	0.5 (0.5-0.5)	0.5 (0.5-1.3)

© 2026 Vasiljevic S et al. *JAMA Network Open*.

Autoantibodies	**PANS** (N = 51)	**Controls** (N = 58)
**S-anti-SS-A (Ro52)** E/mL, reference <1.0		
Above reference, No./total No. (%)	0/44 (0.0)	0/55 (0.0)
Median (range)	0.5 (0.5-0.5)	0.5 (0.5-0.5)
**S-anti-SS-A (Ro60)** E/mL, reference <1.0		
Above reference, No./total No. (%)	0/44 (0.0)	0/55 (0.0)
Median (range)	0.5 (0.5-0.5)	0.5 (0.5-0.5)
**S-anti-SS-B** E/mL, reference <1.0		
Above reference, No./total No. (%)	1/44 (2.3)	0/55 (0.0)
Median (range)	0.5 (0.5-2.7)	0.5 (0.5-0.5)
**S-anti-CENPB** E/mL, reference <1.0		
Above reference, No./total No. (%)	0/44 (0.0)	0/55 (0.0)
Median (range)	0.5 (0.5-0.5)	0.5 (0.5-0.5)
**S-anti-Jo-1** E/mL, reference <1.0		
Above reference, No./total No. (%)	0/44 (0.0)	1/55 (1.8)
Median (range)	0.5 (0.5-0.5)	0.5 (0.5-2.2)
**S-anti-dsDNA** IE/mL, reference <10.0		
Above reference, No./total No. (%)	0/45 (0.0)	0/55 (0.0)
Median (range)	0.5 (0.5-5)	0.5 (0.5-5)
**Thyroid**	**PANS** (N = 51)	**Controls** (N = 58)
**S-TSH** mE/L, reference 0.6-4.8		
Below reference, No./total No. (%)	0/45 (0.0)	2/55 (3.6)
Above reference, No./total No. (%)	1/45 (2.2)	0/55 (0.0
Median (range)	1.8 (1.0-7.1)	2.2 (0.7-8.0)
**S-T4** pmol/L, reference 13-22		
Below reference, No./total No. (%)	3/44 (6.8)	3/54 (5.6)
Above reference, No./total No. (%)	0/44 (0.0)	2/54 (3.7)
Median (range)	16.0 (10.0-21.0)	15.5 (10.0-29.0)
**Other**	**PANS** (N = 51)	**Controls** (N = 58)
**S-25(OH)D** nmol/L, reference 50-250		
Below reference, No./total No. (%)	6/44 (13.6)	14/55 (25.5)
Above reference, No./total No. (%)	0/44 (0.0)	0/55 (0.0)
Median (range)	63.0 (41.0-142.0)	60.0 (28.0-103.0)
**P-ALT** mikrokat/L, reference <0.52		
Above reference, No./total No. (%)	4/48 (8.3)	3/56 (5.4)
Median (range)	0.3 (0.1-0.9)	0.3 (0.1-0.6)
**Throat culture**	**PANS** (N = 51)	**Controls** (N = 58)
**GAS bacteria** positive/negative		
Positive, No./total No. (%)	5/30 (16.7)[b]	4/47 (8.5)

Abbreviations: **PANS**, Pediatric Acute-Onset Neuropsychiatric Syndrome; **B**, blood; **S**, serum; **P**, plasma.
[a]Laboratory analyses conducted within ±2 months of the initial assessment. Reference ranges presented in the table reflect the most commonly observed values in our PANS cohort, based on data from Karolinska University Hospital. Age- and sex-adjusted reference ranges were applied where available. To allow for statistical summarization of distributions, values below or above detection limits were imputed using half the lower limit or the maximum threshold.
[b]Of which one case was diagnosed with GAS or Group G Streptococcal bacteria

© 2026 Vasiljevic S et al. *JAMA Network Open*.

eFigure 2. Association Between Time From Symptom Onset to Laboratory Testing and Number of Laboratory Abnormalities in the PANS Group

Negative binomial regression examining the association between time from symptom onset to laboratory testing (in years) and the total number of immune-related laboratory abnormalities per individual across 56 variables in the PANS group (n = 48). A significant inverse association was observed ($P < 0.05$), indicating that shorter time to testing was associated with more abnormalities. The fitted values line illustrates this trend. The model explained 2.95% of the variance (pseudo-R^2 = 0.0295) and was influenced by four outliers, as shown in the figure.

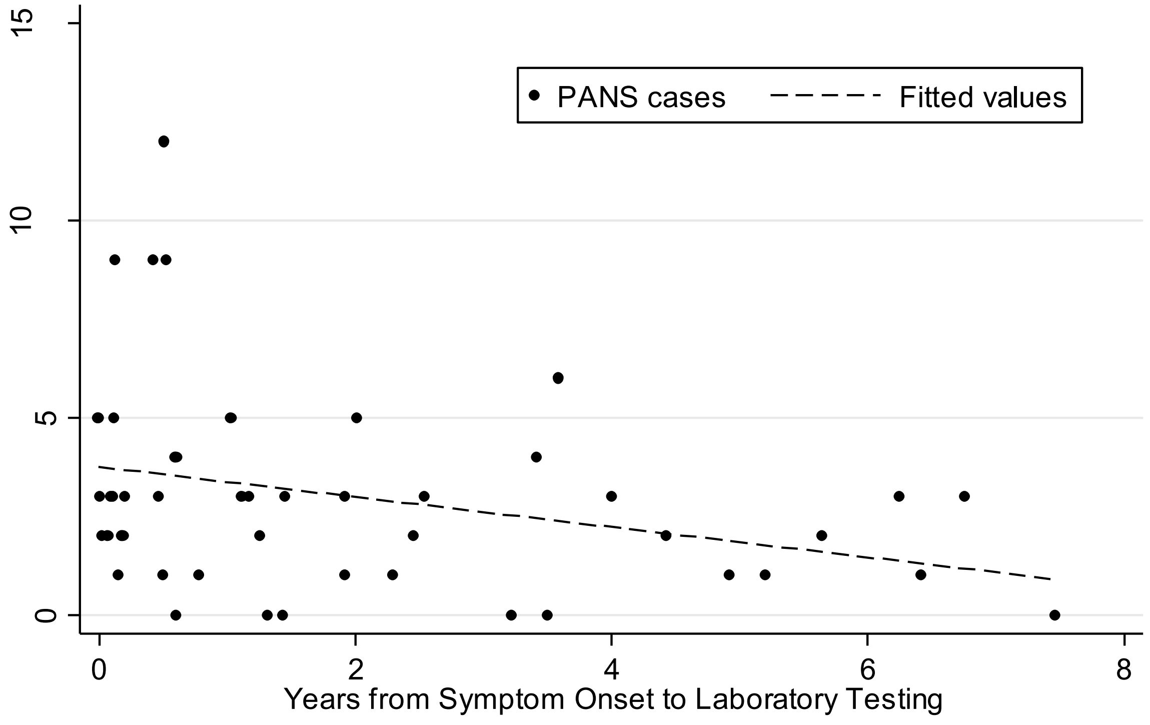

Abbreviations: **PANS**, Pediatric Acute-Onset Neuropsychiatric Syndrome.

© 2026 Vasiljevic S et al. *JAMA Network Open*.

eFigure 3. Principal Component Analysis of Laboratory Variables With Age and Sex as Unbiased Covariates

eFigure 3a. Loadings of Individual Laboratory Variables on the First Two Principal Components

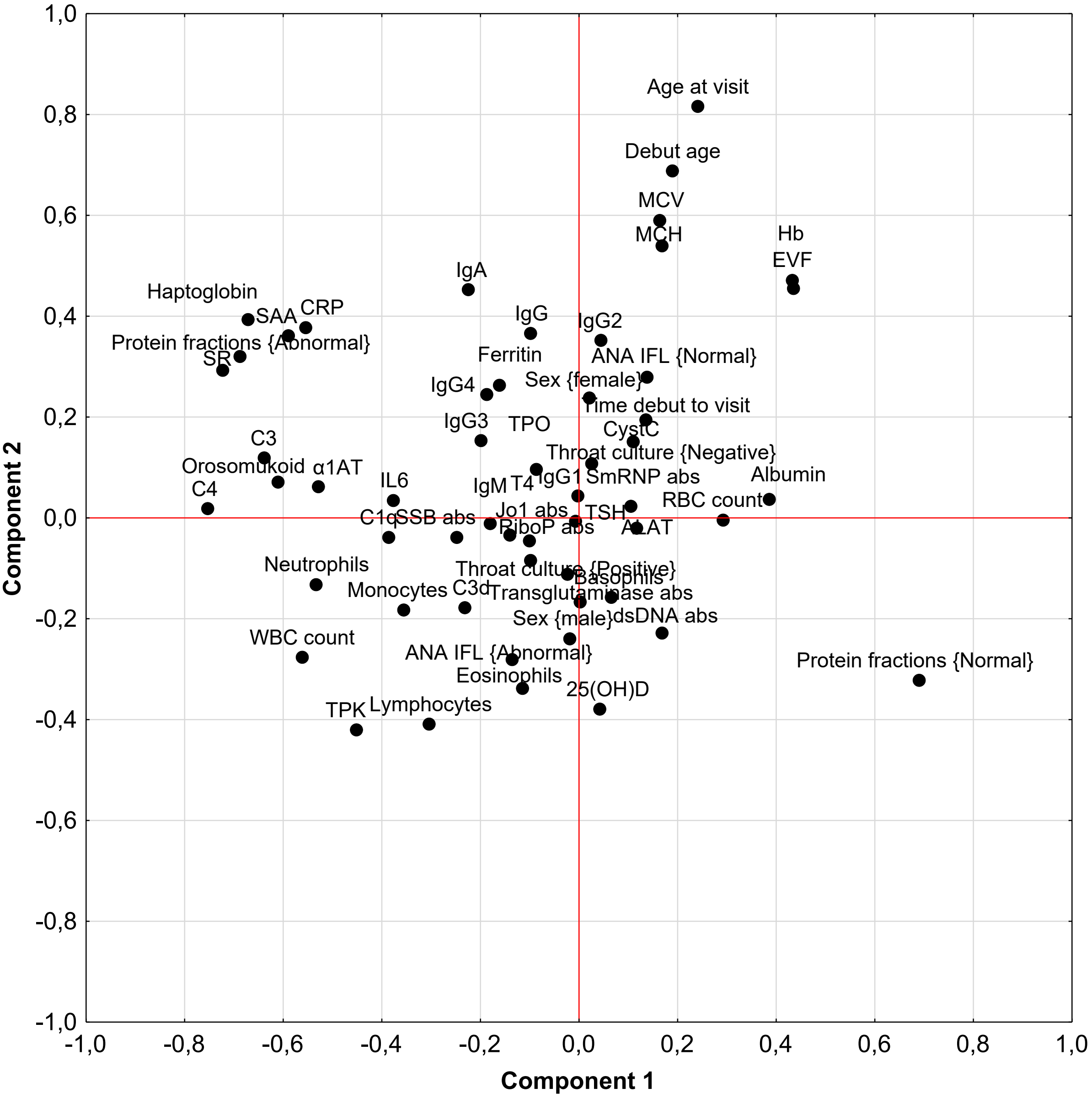

© 2026 Vasiljevic S et al. *JAMA Network Open*.

eFigure 3b. Individual Distributions Across the First Two Principal Components Among PANS Cases (Stars, n = 47) and Controls (Squares, n = 56)

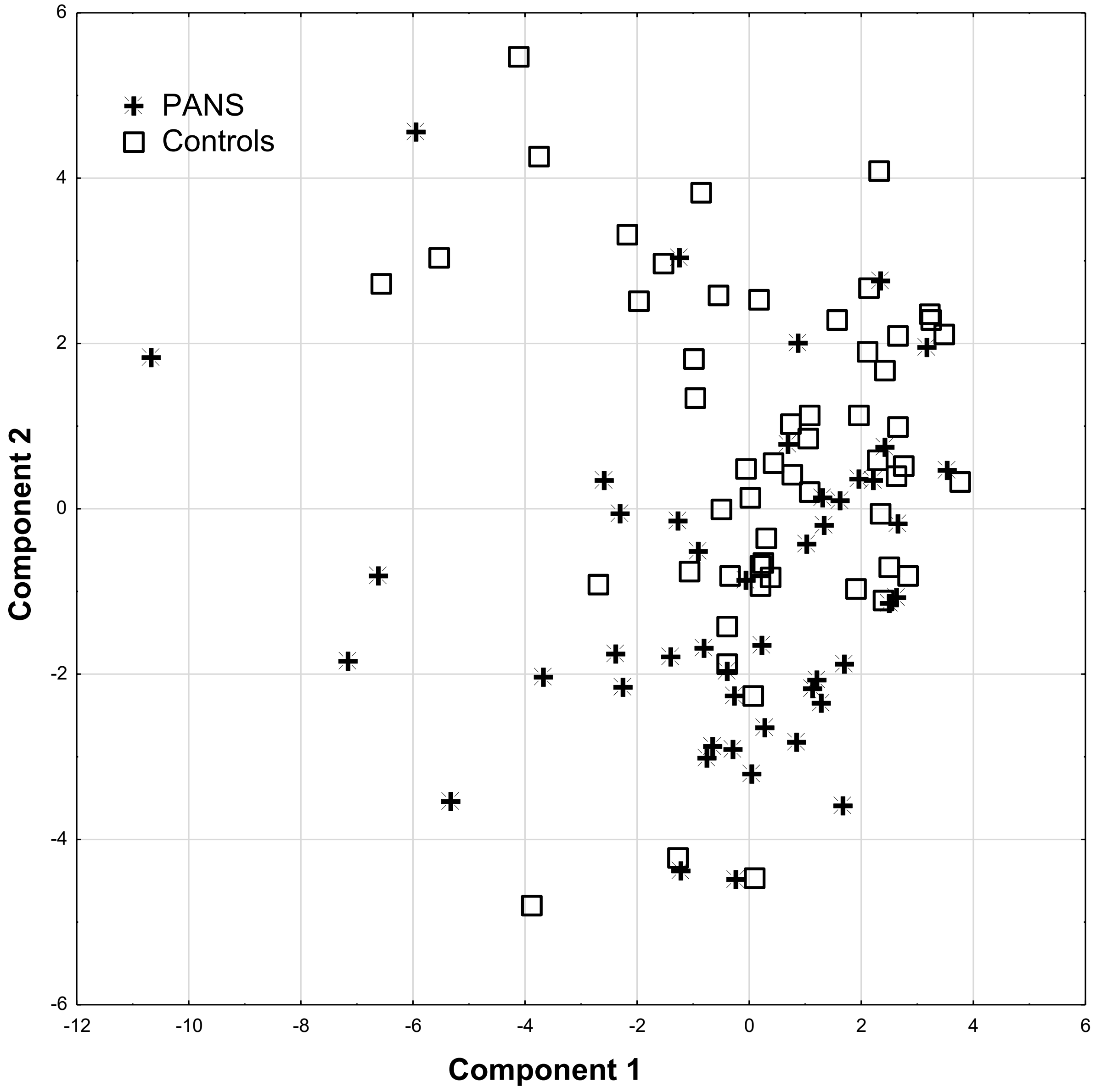

© 2026 Vasiljevic S et al. *JAMA Network Open*.
