# Supplementary material for: Recommended Medical Investigations in Pediatric Acute-Onset Neuropsychiatric Syndrome
Source: JAMA Netw Open. 2026 Mar 20;9(3):e262618. doi: 10.1001/jamanetworkopen.2026.2618 (PMC13005155; doi:10.1001/jamanetworkopen.2026.2618)
Supplement: Supplement 2. — Data Sharing Statement [file jamanetwopen-e262618-s002.pdf]

Data Sharing Statement

Vasiljevic. Reconsidering Recommended Medical Investigations in Pediatric Acute-Onset Neuropsychiatric Syndrome. *JAMA Netw Open*. Published March 20, 2026. doi:10.1001/jamanetworkopen.2026.2618

Data
Data available: No

Additional Information
Explanation for why data not available: Data sharing is not permitted under our ethical approval.
